# Viable *Gata2* deficient mice exhibit T cell development independent of adult definitive hematopoiesis

**DOI:** 10.3389/fimmu.2026.1832484

**Published:** 2026-05-22

**Authors:** Yuhang Li, Shiying Dang, Jinbing Li, Tian Zhang, Juan Du, Guangjin Pan, Ke Huang

**Affiliations:** 1 Guangdong Second Provincial General Hospital, Jinan University, Guangzhou, Guangdong, China; 2 Clinical Medicine Research Institute, The First Affiliated Hospital of Jinan University, Guangzhou, Guangdong, China; 3 Guangzhou Institutes of Biomedicine and Health, Chinese Academy of Sciences, Guangzhou, Guangdong, China

**Keywords:** definitive hematopoiesis, *Gata2*, HSC, knockout, T cells

## Abstract

*Gata2* is indispensable for hematopoiesis, with knockout mice typically exhibiting embryonic lethality attributed to severe anemia and defective hematopoietic stem cell (HSC) development. Intriguingly, we obtained viable adult *Gata2^-/-^* mice harboring a 17-nucleotide deletion in exon 2, which displayed intact T-cell development despite a significantly reduced bone marrow HSC pool. While mutant HSCs exhibited attenuated lymphoid potential, they retained partial myeloid reconstitution capacity. Single-cell transcriptomic analysis revealed compensatory upregulation of key hematopoietic regulators, including *Fos* and *Klf6*, in *Gata2*-deficient HSCs. Comprehensive profiling further demonstrated preservation of the complete T-cell hierarchy, including all major subsets, in *Gata2* mutant mice. These findings provide evidence that *Gata2* knockout mice may be viable, and T-cell development may proceed independently of definitive hematopoiesis.

## Highlights

- A rare subset of *Gata2* knockout mice survived to adulthood, providing a unique model to study Gata2-independent hematopoiesis.
- *Gata2* knockout mice showed normal T cell development despite severe HSC depletion and B cell deficiency.

## Introduction

*Gata2*, encoding a zinc finger transcription factor, is a master regulator of both primitive and definitive hematopoiesis. Germline deletion of *Gata2* in mice causes embryonic lethality at embryonic day 10-10.5 (E10-E10.5), characterized by severe anemia and reduced yolk sac hematopoietic progenitor cells (HPCs) (1). Furthermore, endothelial-specific conditional knockout models have revealed that *Gata2* is indispensable for hematopoietic stem cell (HSC) generation in the aorta-gonad-mesonephros (AGM) region and subsequent HSC maintenance, with embryonic lethality occurring at E14 and accompanied by fetal liver (FL) anemia (2). Deletion of the *Gata2* cis-regulatory element (+9.5) also completely abrogates HSC generation in the AGM region, causing embryonic lethality at E13-E14 (3).

Our previous work using *GATA2* knockout human embryonic stem cells (hESCs) demonstrated impaired endothelial-to-hematopoietic transition (EHT) and defective HPC production (4), findings that were independently confirmed (5). Interestingly, a *Gata2Venus* reporter system identified *Gata2*-independent HPCs in the aorta, vitelline and umbilical arteries, and fetal liver, which exhibited unique functional properties (6). However, whether *Gata2*-independent HPCs possess lymphocyte differentiation potential remains unknown. Notably, an immune-restricted progenitor with lymphoid potential was identified prior to definitive HSC formation (7).

Despite these advances, the precise role of *GATA2* in lymphocyte development remains unclear due to embryonic lethality. Recent mouse studies showed that *Gata2* + 9.5 enhancer mutations disrupt adult HSC self-renewal and T-cell differentiation (8). Conversely, zebrafish *gata2b-*deficient mutants exhibited viability but showed reduced hematopoietic stem and progenitor cells (HSPCs) and myeloid progenitors, coupled with an unexpected expansion of lymphoid progenitors—specifically B cells, not T cells (9). However, limitations in zebrafish transplantation assays preclude resolving whether *gata2b*-deficient T cells originate from embryonic HPCs or definitive HSPCs. These gaps underscore the need for a viable *Gata2* mutant mouse model to systematically investigate the role of *Gata2* in lymphoid lineage specification and the hematopoietic hierarchy.

Here, we report the unexpected viability of a subset of *Gata2* knockout mice harboring a 17-nucleotide deletion in exon 2, which survive to adulthood despite profound hematopoietic defects. These mutant mice exhibit a complex hematopoietic phenotype characterized by: (1) normal peripheral T-cell proportions but reduced B-cell frequencies accompanied by monocytosis and granulocytosis; (2) markedly decreased bone marrow hematopoietic stem cells (HSCs) and common lymphoid progenitors (CLPs); and (3) intact thymic T-cell progenitor populations but impaired B-cell progenitor development. Transplantation studies revealed that *Gata2*-deficient HSCs lack long-term repopulation capacity and display skewed differentiation potential with preserved myeloid but compromised lymphoid lineage output. Single-cell transcriptomics identified compensatory upregulation of key hematopoietic regulators, including c-*Fos* and *Klf6*, in mutant HSCs, potentially explaining their survival. Notably, comprehensive transcriptomic analysis further demonstrated largely preservation of the T-cell hierarchy. These findings establish that T-cell progeny may develop independently of definitive hematopoiesis in adult mice, challenging current paradigms of the hematopoietic hierarchy.

## Methods

### Generation of *Gata2* knockout mice

The work has been reported in line with the ARRIVE guidelines 2.0.

The sgRNA targeting *Gata2* Exon2 was designed via CRISPick (10) and synthesized by IGE Biotechnology. The sgRNA and CRISPR/Cas9 mRNA were transcribed *in vitro* using the New England Biolabs (NEB) system.

The *Gata2* knockout mice were generated by microinjecting CRISPR/Cas9 mRNA and sgRNA into C57BL/6 zygotes. Following microinjection, the zygotes were transferred into pseudo-pregnant females. A founder heterozygous *Gata2^+/-^* female, harboring a 17-nucleotide deletion in exon 2, was identified by genotyping and bred with wild-type males to establish a stable *Gata2^+/-^* colony. Homozygous *Gata2^-/-^* mice were obtained via sibling mating, yielding five individuals (one male, four females). Notably, all *Gata2^-/-^* individuals exhibited infertility when intercrossed with wild-type (WT), *Gata2^+/-^*, or *Gata2^-/-^* counterparts, indicating a potential germline transmission defect associated with *Gata2* deficiency. One male and one female *Gata2^-/-^* mouse survived for over one year; the other three were sacrificed for non-competitive/competitive transplantation and single cell RNA-sequencing (scRNA-seq), respectively, including analysis of the bone marrow (BM), thymus, and spleen.

For euthanasia, mice were placed in a chamber, and 100% CO2 was introduced at a fill rate of 30-70% displacement of the chamber volume per minute, with CO2 added to the existing air in the chamber. Animals were exposed to CO2 until complete cessation of breathing was observed for at least 2 minutes (5–10 minutes may be required). Animals were visually inspected to confirm the absence of movement and respiration.

Genotyping was confirmed by Sanger sequencing. Genomic DNA from was extracted toe biopsies (or indicated organs) using a commercial DNA purification kit (TIANGEN). The target region flanking the sgRNA site was PCR-amplified with high-fidelity DNA polymerase (Vazyme) and sequenced. Primers used were:.

F: 5’-CGCGAGTTTCCCTGCAAGTGTATGAG-3’.

R: 5’-CCCTGCGAGTCGAGATGGTTGAAG-3’.

### Hematopoietic stem cell transplantation in mice

For non-competitive primary transplantation, BM cells were flushed from the femurs of WT, *Gata2^+/-^* and *Gata2^-/-^* mice (CD45.2^+^) with ice-cold PBS using a syringe, followed by red blood cell (RBC) lysis to enrich mononuclear cells. Recipient mice (CD45.1^+^) received split-dose irradiation (2 × 4.45 Gy, 6 hours apart) for myeloablation, followed by retro-orbital venous injection of 0.5 million donor cells for hematopoietic reconstitution. For non-competitive secondary transplantation, BM cells were harvested from primary recipients, isolated and processed as described above.

For competitive primary transplantation, CD45.2^+^ BM cells from the femurs of WT, *Gata2^+/-^*, and *Gata2^-/-^* mice were mixed at a 1:1 ratio (0.25 million cells each) with CD45.1^+^ BM from the femurs of WT C57BL/6 mice, followed by transplantation into irradiated recipients as above. For competitive secondary transplantation, 1 million BM cells were isolated from primary recipients of the competitive transplantation and processed identically.

### Flow cytometry analysis of mouse blood cells

For peripheral lineage analysis, peripheral blood was collected from the orbital vein into sodium citrate, subjected to RBC lysis, and stained with lineage-specific antibodies for flow cytometry. CD45.1-FITC (BioLegend 110706) and CD45.2-PE (BioLegend 109808) were used to distinguish donor origins. Erythroid cells were labeled with Ter119 (BioLegend 116227), T cells with CD90.2-APC (BioLegend Cat: 105311), B cells with CD19-PE/Cy7 (BioLegend 115519), myeloid cells with CD11b-AF700 (BioLegend 101222), and granulocytes/monocytes with Gr1-APC/CY7 (BioLegend 108424). DAPI was used to exclude dead cells.

Hematopoietic stem and progenitor cell analysis: Following euthanasia, BM cells were harvested, subjected to RBC lysis, and analyzed by flow cytometry; the spleen and thymus were isolated, homogenized, filtered through a 70-µm mesh to obtain a single-cell suspension for flow cytometry analysis. The flow cytometry panels used were as follows: LT-HSCs: lineage (CD2/CD3/CD4/CD8/B220/CD11b/Gr1/Ter119/CD48)-biotin, streptavidin-BV750, CD34-FITC, Sca1-Alexa Fluor700, c-Kit-APC/Cy7, CD150-PE/Cy7, CD135-PE, CD45.1-PerCP/Cy5.5, CD45.2-APC; MPP subtypes: lineage (CD2/CD3/CD4/CD8/B220/CD11b/Gr1/Ter119)-biotin, streptavidin-BV750, CD48-FITC, Sca1-Alexa Fluor700, c-Kit-APC/Cy7, CD150-PE/Cy7, CD135-PE, CD45.1-PerCP/Cy5.5, CD45.2-APC; B cell progenitors (BM/spleen): lineage (CD2/CD3/CD4/CD8/Ter119/Mac1/Gr1/NK1.1/IgM/IgD)-APC, B220-FITC, CD43-PE, Ly51-PE/Cy7, CD24-AF700, CD45.2-Alex700; Cell cycle: lineage (CD3/B220/CD11b/Gr1/Ter119/CD48)-FITC, Ki67-APC, c-Kit-APC/Cy7, Sca1-PerCP/Cy5.5, CD150-PE/Cy7, CD135-PE, CD34-AF700, DAPI; Apoptosis: lineage (CD3/B220/CD11b/Gr1/Ter119/CD48)-FITC, ANNEXIN V-APC, c-Kit-APC/Cy7, Sca1-PerCP/Cy5.5, CD150-PE/Cy7, CD135-PE, CD34-AF700, DAPI; Thymus T progenitors and T cells: CD4-APC/CY7, CD8-PE, CD44-PE/CY7, CD25-Percp/Cy5.5, CD45.2-APC. All flow cytometry analyses were performed on a Beckman CytoFLEX S instrument, with DAPI used to discriminate between live and dead cells.

### WBC, RBC, and PLT cell counts

Blood was collected from the mouse orbit using sodium citrate as an anticoagulant, and WBC, RBC, and PLT counts were measured with a Sysmex XN-1000V automated hematology analyzer.

### Isolation of Sca1^+^ bone marrow cells for single-cell RNA sequencing

BM cells were flushed from mouse femurs with cold PBS. The Sca-1^+^ cells were isolated by magnetic-activated cell sorting (MACS) using a biotin-conjugated anti-Sca-1 antibody and streptavidin-coated microbeads (Miltenyi Biotec). The enriched Sca-1^+^ cells were used for scRNA-seq, with libraries prepared using the DNBelab C Series Single-Cell Library Prep Set (MGI, #1000021082) according to the manufacturer’s protocol. The raw sequencing data are stored in National Genomics Data Center (https://ngdc.cncb.ac.cn) under the GSA accession number CRA028125.

### Single-cell RNA-seq data analysis

Raw sequencing data were preprocessed using BGI PISA v2.1 (11). This included filtering low-quality reads, aligning cDNA reads to the mouse genome (mm10), annotating genes, identifying cell-containing beads via UMI counts, merging cDNA reads, and generating a gene expression matrix. Downstream analysis was performed using Seurat v4.2.0 (12) in R. Cells expressing ≥200 genes and ≥500 UMIs with <25% mitochondrial RNA were retained; genes detected in fewer than three cells were removed. Potential doublets were identified and removed using DoubletFinder v2.0.3 (13). Data were normalized (method = “LogNormalize”, scale factor = 10,000), scaled, and subjected to PCA (top 50 PCs); UMAP used dimensions 1–50. Cell cycle phases (G1, S, G2/M) were assigned by the cyclone function in scran v.1.18.7 (14). FindMarkers was used to identify differentially expressed genes (DEGs), followed by KEGG and GO enrichment via DAVID (15). Group proportions per cluster were calculated in R and visualized using ggplot2.

### Gene set enrichment analysis for pathway enrichment

DEGs between groups were identified using the FindMarkers function (only.pos = FALSE and logfc.threshold = 0) and ranked by avg_log2FC. Ranked genes were used as input for GSEA with the clusterProfiler v3.16.0 (16) R package, using gene sets from the MSigDB (17) to assess pathway and gene set enrichment across groups.

### Transcription factor regulatory network analysis

Mouse TF-target interaction data were obtained from the TRRUST (18) database. High-expression transcription factors (avg_log2FC > 0.25, p_val < 0.05) were screened from DEGs of the three groups (WT, *Gata2^+/−^*, and *Gata2^-/-^*), and target genes labeled as “Activation” in the database were selected. The TF-target regulatory network was visualized using Cytoscape (19).

### SCENIC analysis of the tHSC1 subset

SCENIC analysis was performed on the tHSC1 subset of the scRNA-seq data to infer transcription factor-centered regulatory networks. Raw UMI counts from tHSC1 cells were extracted from the Seurat object and analyzed using the pySCENIC (20) pipeline. Candidate TF–target interactions were first inferred by gene regulatory network analysis, followed by cisTarget-based motif enrichment pruning to identify motif-supported regulons. Regulon activity in individual cells was quantified using AUCell. Regulon activity scores were imported into R for downstream visualization.

## Results

### Identification of viable *Gata2*-deficient mice and analysis of their peripheral hematopoietic profile

**Figure 1:**
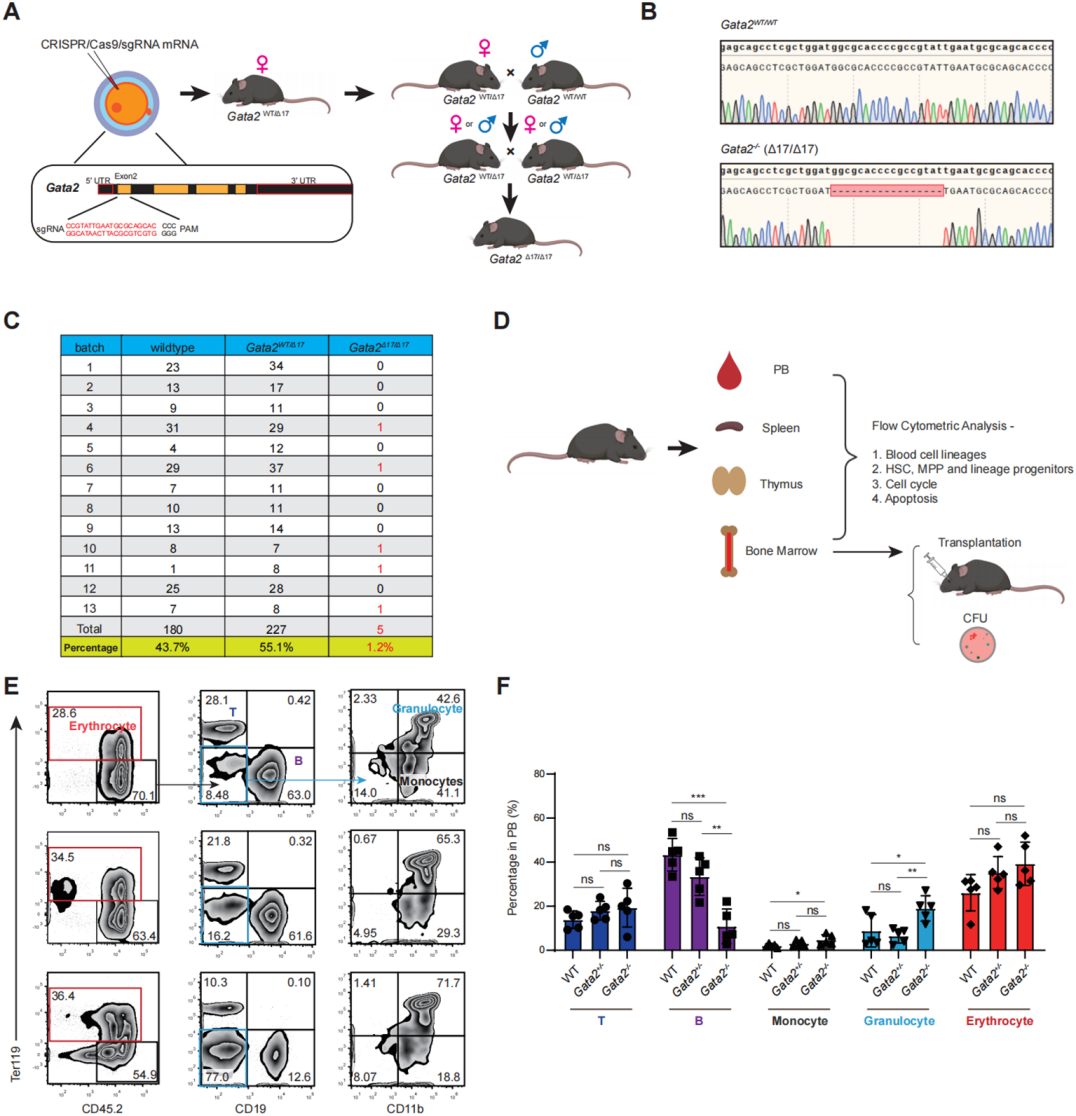
Establishment of viable *Gata2*-deficient mice and analysis of peripheral hematopoietic profiles. **(A)** Schematic representation of the experimental strategy for generating *Gata2-*deleted mice. **(B)** Representative chromatograms showing the genomic sequences of wild-type and *Gata2-*deleted mice. **(C)** Distribution of genotypes among newborn mice, including wild-type, heterozygous, and homozygous *Gata2-*deleted individuals. **(D)** Illustration of the experimental procedure for the indicated analyses. Flow cytometry analysis **(E)** and statistics **(F)** of hematopoietic cell lineages in the peripheral blood of the WT, *Gata2^+/-^* and *Gata2^-/-^* mice. n = 5 individual mice of one experiment. *Error bars* represent mean ± S.D. *p<0.05; **p<0.01; ***p<0.001; ns, not significant.

We first generated a *Gata2^+/-^* mouse line using CRISPR/Cas9 by introducing a 17-nucleotide deletion in Exon 2 of the *Gata2* locus (**Figure 1A**), resulting in a premature stop codon and a truncated, nonfunctional protein (**Supplementary Figure 1A**). The *Gata2^+/-^* mice were subsequently bred to generate germline *Gata2^-/-^* mouse (**Figure 1A**). Survival analysis of 13 litters revealed a strikingly low survival rate among *Gata2^-/-^* mice. Of the 412 neonates genotyped, only 5 *Gata2^-/-^* pups survived (1.2%), far below the expected Mendelian ratio (**Figure 1B**). Homozygous deletion was confirmed by PCR-based genotyping and Sanger sequencing of tail-derived genomic DNA (**Figure 1C**), with tissue-specific validation from peripheral blood mononuclear cells (PBMCs) and multiple organs, including the kidney, heart and lung (**Supplementary Figure 1B**).

Given the well-documented hematopoietic deficiencies associated with *Gata2* deficiency, we analyzed blood cell lineages and their progenitors in viable *Gata2^-/-^* mice (**Figure 1D**). Complete blood count analysis revealed normal levels of white blood cells (WBCs), red blood cells (RBCs), and platelets (PLTs) in *Gata2^-/-^* mice compared to wild-type and *Gata2^+/-^* controls (**Supplementary Figure 1C**). Further characterization of PBMCs demonstrated that viable *Gata2^-/-^* mice maintained normal proportions of Ter119^+^ erythrocytes and Ter119^-^CD45.2^+^CD90.2^+^ T cells (**Figure 1E**). However, these mice exhibited significant alterations in other lineages, characterized by reduced Ter119^-^CD45.2^+^CD19^+^ B cells and increased Ter119^-^CD45.2^+^CD11b^+^Gr1^-^ monocytes and Ter119^-^CD45.2^+^CD11b^+^Gr1^+^ granulocytes (**Figure 1F**). These findings suggest a distinct lineage bias in *Gata2*-deficient hematopoiesis.

### Impaired hematopoietic stem cell compartment in viable Gata2 knockout mice

**Figure 2:**
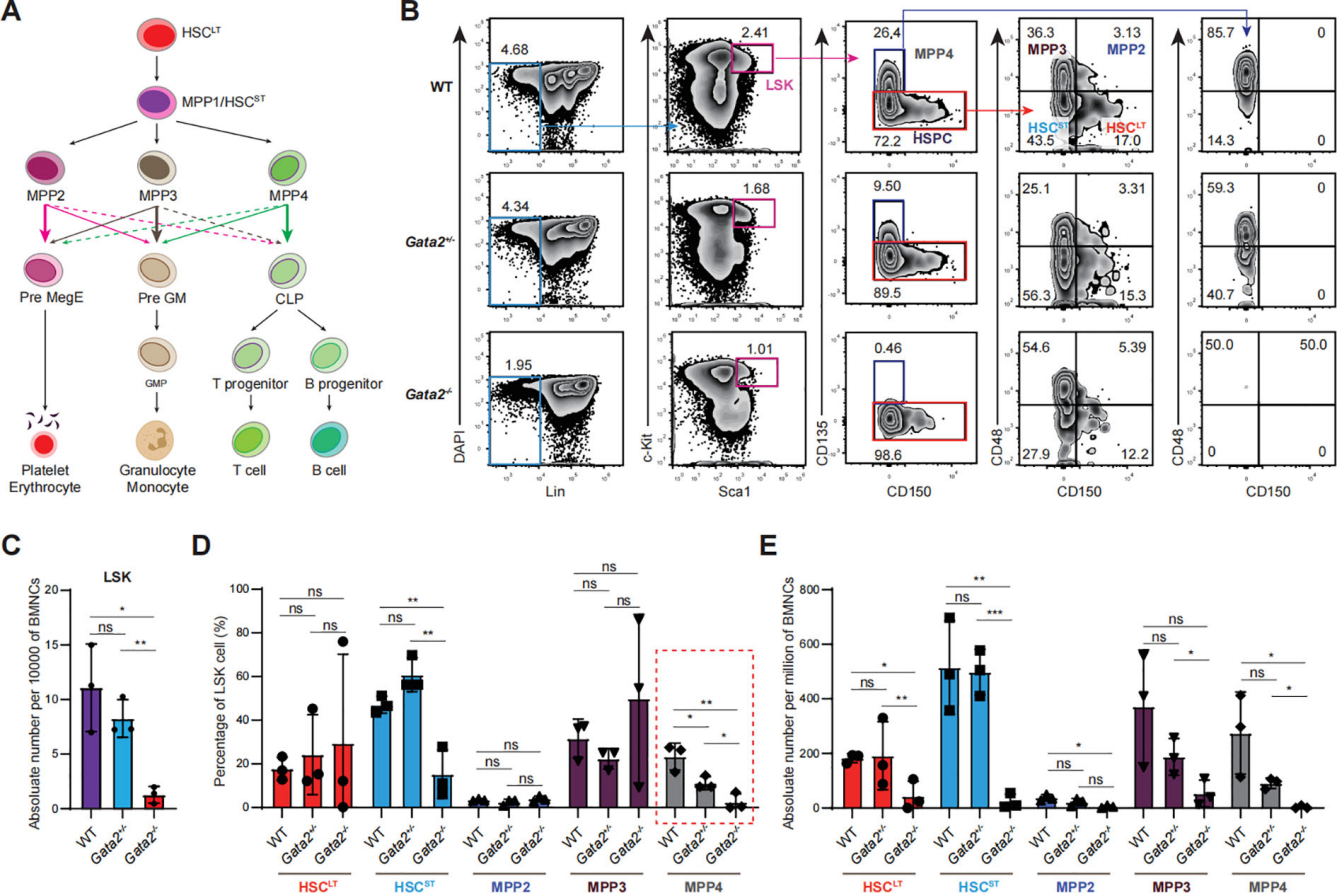
Impaired hematopoietic stem cell compartment in viable *Gata2* knockout mice. **(A)** The classical hematopoietic hierarchy. **(B)** Flow cytometry analysis of Lin^-^Sca1^+^c-Kit^+^ (LSK) HSPC subsets, including HSC^LT^, HSC^ST^, MPP2, MPP3, and MPP4 in the indicated groups. **(C)** Quantification of LSK HSPC cell numbers in WT, *Gata2^+/−^* and *Gata2^−/−^* mice. **(D)** Quantification of the percentages of HSPC subsets (HSC^LT^, HSC^ST^, MPP2, MPP3, and MPP4) in the indicated groups. **(E)** Quantification of HSPC subset numbers in the indicated groups. n = 3 independent individual mice of one experiment. Statistical analysis was performed using unpaired two-tailed Student’s t-test. Data are presented as mean ± SD. *p<0.05; **p<0.01; ns, no significance.

To further investigate the impact of *Gata2* deficiency on hematopoiesis, we analyzed the HSPC compartment in the bone marrow (BM) of *Gata2^-/-^* mice versus wild-type and *Gata2^+/-^* controls. Within the classical hematopoietic hierarchy (**Figure 2A**), LSK HSPCs encompass CD135^-^CD150^+^CD48^-^ long-term HSCs (HSC^LT^), which differentiate into CD135^-^CD150^-^CD48^-^ short-term HSCs (HSC^ST^) or multipotent progenitors 1 (MPP1). These HSC^ST^/MPP1 cells subsequently give rise to CD135^-^CD150^+^CD48^+^ MPP2, CD135^-^CD150^+^CD48^-^ MPP3, and CD135^+^CD150^-^CD48^+^ MPP4 subsets (**Figure 2B**) (21). MPP2 cells primarily generate megakaryocyte/erythroid lineages and MPP3 cells contribute to granulocyte/monocyte differentiation, while MPP4 cells predominantly produce CLPs that develop into T and B lymphocytes.

While *Gata2^-/-^* mice exhibited comparable numbers of bone marrow mononuclear cells (BMNCs) (**Supplementary Figure 2A**), LSK HSPCs were markedly reduced(**Figure 2C**). Although the relative frequencies of HSPC subsets displayed heterogeneous changes (**Figure 2D**), the absolute numbers of all progenitor populations were uniformly reduced in *Gata2^-/-^* mice (**Figure 2E**). This observation was further confirmed using an alternative HSPC classification system based on CD135 and CD34 expression, defining HSC^LT^(CD135^-^CD34^-^), HSC^ST^ (CD135^-^CD34^+^), and MPPs (CD135^+^CD34^+^) within the LSK population (**Supplementary Figure 2B**) (22). Consistent with previous findings, all three subsets were significantly diminished in *Gata2^-/-^* mice (**Supplementary Figure 2C**). However, while previous studies have demonstrated that *Gata2* deficiency leads to impaired survival of HSCs, the viable *Gata2*-mutant mice in our study exhibited normal HSC proliferation and apoptosis (**Supplementary Figures 2D–F**), along with a modest colony-forming capacity, which aligns with the observed reduction in HSPC generation (**Supplementary Figure 2G**). These results collectively demonstrate that *Gata2* deficiency leads to a substantial reduction in the HSC and progenitor pool and disrupts the differentiation into hematopoietic progenitor cells.

### The viable *Gata2* mutant mice demonstrate a reduction in B-cell progenitors while maintaining normal levels of T-cell progenitors

**Figure 3:**
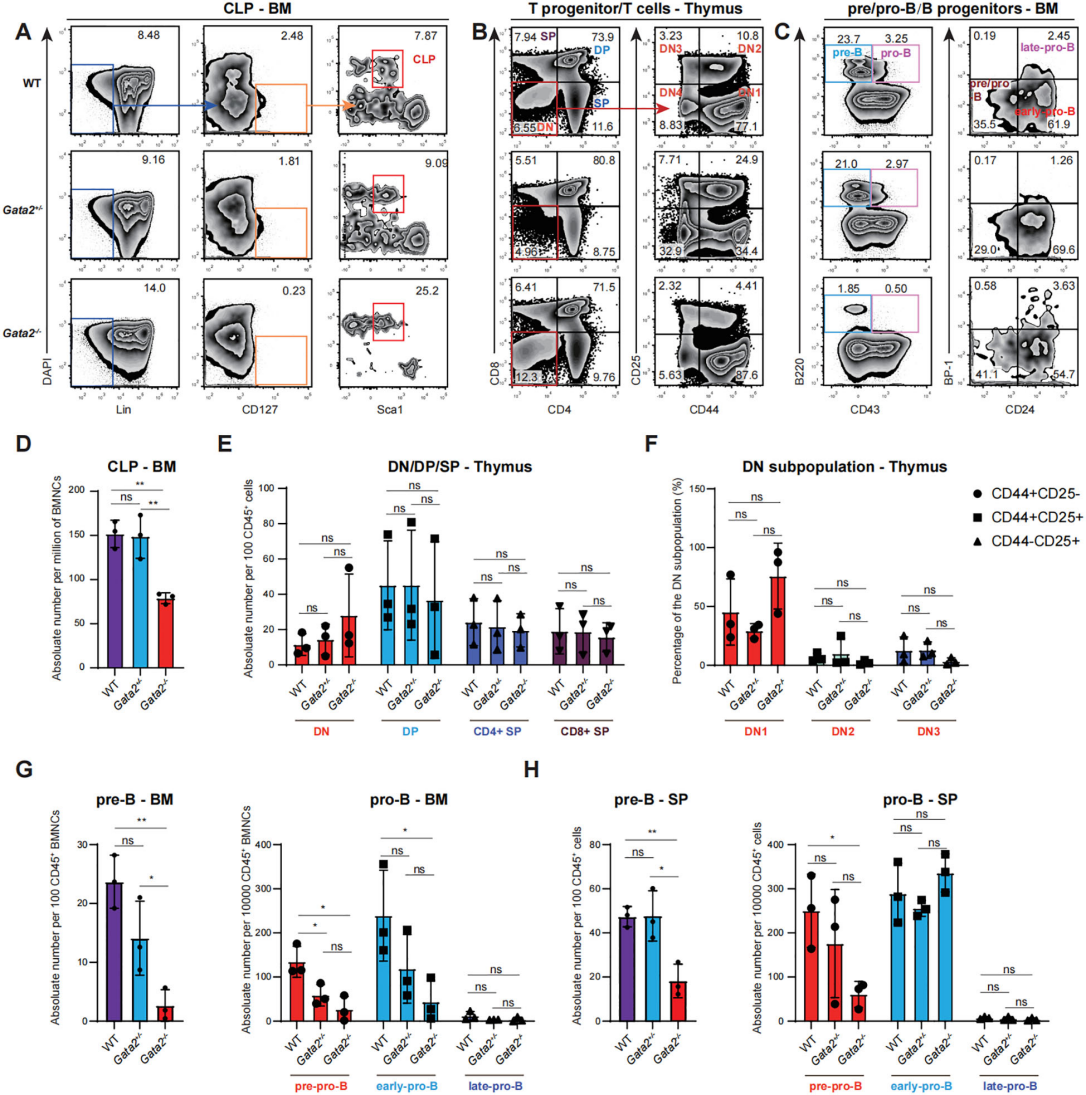
Analysis of hematopoietic cells in bone marrow, thymus and spleen of the indicated groups. **(A)** Flow cytometry analysis of the Lin^-^CD127^+^CD11b^-^c-Kit^+^Sca1^low^ common lymphoid progenitor (CLP) in the bone marrow of WT, *Gata2^+/−^* and *Gata2^−/−^* mice. **(B)** Flow cytometric analysis of the T cell progenitors and populations in the thymus (CD45.2^+^ gated), including the CD4^−^CD8^−^ double-negative (DN), CD4^+^CD8^+^ double^-^positive (DP), CD4^+^ single-positive (CD4^+^ SP), and CD8^+^ single-positive (CD8^+^ SP) populations in the indicated mice. DN subsets were further classified into CD44^+^CD25^-^ DN1, CD44^+^CD25^-^ DN2, CD44^+^CD25^-^ DN3, CD44^+^CD25^-^ DN4 subpopulations. **(C)** Flow cytometric analysis of the B cell progenitors in the bone marrow (CD45.2^+^Lin- gated), including B220^+^CD43^-^ pre-B cells and B220^+^CD43^+^ pro-B cells. The pro-B cells were further divided into BP-1^-^CD24^-^ pre-pro-B cells, BP-1^-^CD24^+^ early pro-B cells and BP-1^+^CD24^+^ late-pro-B cells. **(D)** Quantification of common lymphoid progenitor (CLP) cell numbers in bone marrow of the indicated groups. **(E)** Quantification of T cell progenitor numbers in the indicated groups. **(F)** Quantification of DN subset percentages in the indicated groups. **(G)** Quantification of pre-B and pro-B cell number in the bone marrow of the indicated groups. **(H)** Quantification of pre-B and pro-B cell number in the spllen of the indicated groups. n = 3 independent individual mice of one experiment. Statistical analysis was performed using unpaired two-tailed Student’s t-test. Data are presented as mean ± SD. *p<0.05; **p<0.01; ns, no significance.

As *Gata2* deficiency led to significant down-regulation of HSCs and MPPs, particularly MPP4, we investigated its downstream effects on lymphoid progenitor development (**Supplementary Figure 3A**), including Lin^-^CD127^+^CD11b^-^c-Kit^+^Sca1^low^ CLPs in the BM (**Figure 3A**), thymic T progenitors(**Figure 3B**), and B cell progenitors in the BM and spleen (**Figure 3C**). In the thymus, T cells are categorized by CD4/CD8 expression (23) into CD4^-^CD8^-^ double-negative (DN), CD4^+^CD8^+^ double-positive (DP), CD4^+^ single-positive (CD4^+^ SP), and CD8^+^ single-positive (CD8^+^ SP) subsets. The DN population is further subdivided into four stages: CD44^+^CD25^−^ DN1, CD44^+^CD25^+^ DN2, CD44^−^CD25^+^ DN3, and CD44^−^CD25^−^ DN4. In the BM, B cell progenitors are classified into B220^+^CD43^-^ pre-B cells and B220+CD43+ pro-B cells. The pro-B cells can be further divided into BP-1-CD24- pre-pro-B cells, BP-1-CD24+ early-pro-B cells, and BP-1+CD24+ late-pro-B cells (**Figure 3C**) (24).

We observed a significant reduction in CLPs in the BM of *Gata2^-/-^* mice (**Figure 3D**). However, thymus composition remained unaltered, with unchanged DN, DP, CD4^+^ SP, and CD8^+^ SP cell frequencies (**Figure 3E**). Furthermore, a detailed examination revealed that the percentage of individual DN subsets was unaffected by the *Gata2* mutation (**Figure 3F**). These results indicate that the development of T cell lineages remains unaffected by *Gata2* deficiency despite the significant reduction in CLPs. Conversely, *Gata2* knockout markedly decreased B cell progenitors in the BM, including pre-B cells, pre-pro-B cells, and early-pro-B cells, while late-pro-B cells did not exhibit significant alterations, potentially attributable to their rarity within the total cellular population (**Figure 3G**). Interestingly, *Gata2*-deficient mice displayed an increase in spleen weight (**Supplementary Figures 3B, C**). Given the spleen’s pivotal role in B cell development, we conducted a comprehensive analysis of splenic B progenitors (**Supplementary Figure 3D**). Similar to the findings in the BM, the absence of *Gata2* led to a pronounced reduction in pre-B cells and pre-pro-B cells, while early-pro-B cells and late-pro-B cells remained relatively stable (**Figure 3H**), suggesting that the spleen may employ compensatory mechanisms to mitigate the developmental abnormalities in B cells resulting from Gata2 deficiency. Nevertheless, these results suggest that *Gata2* knockout leads to a reduction in the generation of CLPs and significantly impairs B cell development, while maintaining T lineage development.

### *Gata2* mutated HSCs showed impaired long-term repopulating capacity in serial non-competitive transplantation assay

**Figure 4:**
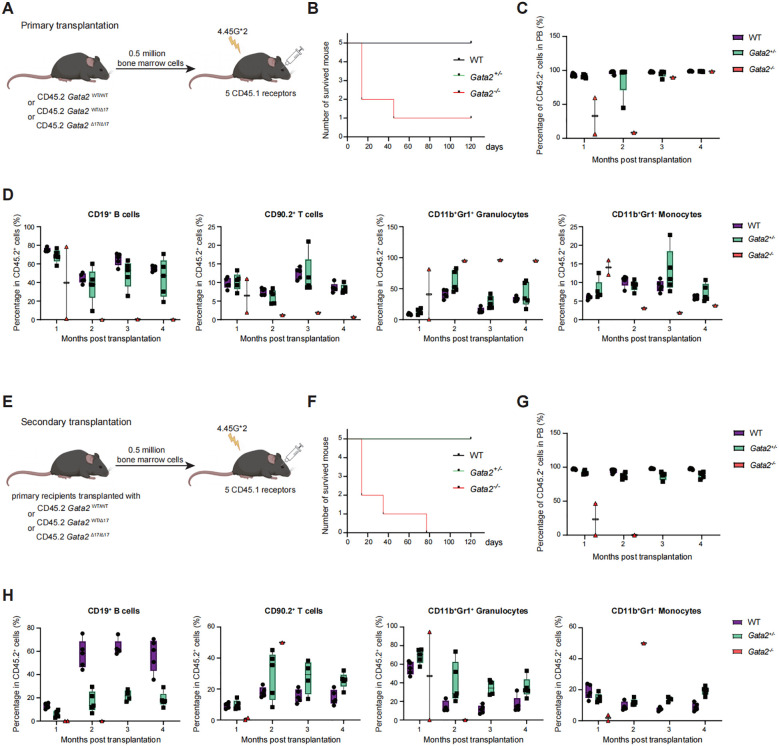
Detection of hematopoietic cells generated in non-competitive transplantation. **(A)** Scheme of the primary non-competitive transplantation experiment. **(B)** Survival curves of recipients transplanted with WT, *Gata2^+/-^*, *Gata2^-/-^* bone marrow cells. **(C)** Quantification of CD45.2^+^ donor-derived cells in the peripheral blood of recipient mice. Wild-type and *Gata2^+/-^* group: n = 5. *Gata2^-/-^* group: n = 2 for month 1, n = 1 from month 2 to 4. **(D)** Percentages of CD19^+^ B cells, CD90.2^+^ T cells, CD11b^+^Gr1^+^ granulocytes, and CD11b^+^Gr1^+^ monocytes among CD45.2^+^ engrafted cells in the peripheral blood of transplanted mice. **(E)** Schematic representation of secondary transplantation for the indicated groups. **(F)** Survival curves of secondary transplantation recipients transplanted with the bone marrow cells derived from primary recipients. **(G)** Quantification of CD45.2^+^ donor-derived cells in the peripheral blood of secondary transplanted mice. Wild-type and *Gata2^+/-^* groups: n = 5. *Gata2^-/-^* group: n = 2 for month 1, and n = 1 for month 2. No mice survived beyond 3 months. **(H)** Percentages of CD19^+^ B cells, CD90.2^+^ T cells, CD11b^+^Gr1^+^ granulocytes, and CD11b^+^Gr1^+^ monocytes among CD45.2^+^ engrafted cells in the peripheral blood of the secondary transplanted mice.

To investigate the impact of *Gata2* mutation on the long-term repopulating capacity of HSCs, we performed serial non-competitive transplantation assays. WT, *Gata2^+/−^*, and *Gata2^−/−^* BM cells (CD45.2+) were transplanted into lethally irradiated recipient mice (CD45.1+). All five recipient mice received bone marrow cells derived from a single donor mouse (**Figure 4A**). Only two recipients transplanted with *Gata2^−/−^* BM cells survived beyond 1 month, and one survived until 4 months (**Figure 4B**). In these survivors, host-derived cells persisted for two months post-transplantation; however, by the third month, all detectable cells were donor-derived (**Figure 4C**), demonstrating that *Gata2^−/−^* HSCs retain a reduced but robust hematopoietic reconstitution potential in primary recipients. Furthermore, peripheral blood analysis revealed a reduced proportion of donor-derived B and T cells but an elevated granulocyte contribution, indicative of a myeloid lineage bias in *Gata2*-mutated HSCs (**Figure 4D**).

BM cells from primary recipients sacrificed at 4 months post-transplantation were transplanted into secondary recipients. All five recipient mice received bone marrow cells derived from a single donor mouse (**Figure 4E**). Secondary recipients of *Gata2^-/-^* BM exhibited rapid mortality, with only one mouse surviving beyond 2 months (**Figure 4F**). This survivor displayed minimal donor-derived chimerism by the second month (**Figure 4G**). Consistent with primary recipients, granulocytes dominated donor-derived populations during the first month in surviving secondary recipients (**Figure 4H**). Intriguingly, the single secondary recipient surviving into the second month exhibited a chimeric population dominated by T cells and monocytes despite overall low donor chimerism (**Figure 4H**). This mouse manifested severe radiation-induced pathology, including cutaneous desquamation and necrosis. Given the established role of hematopoietic stem cell transplantation in mitigating radiation injury, these findings imply an intrinsic lack of HSC-mediated radioprotection and deficiency of *Gata2^-/-^* HSCs.

### *Gata2*-deficient HSCs exhibited defective long-term reconstitution capacity but myelopoiesis in serial competitive transplantation assays

**Figure 5:**
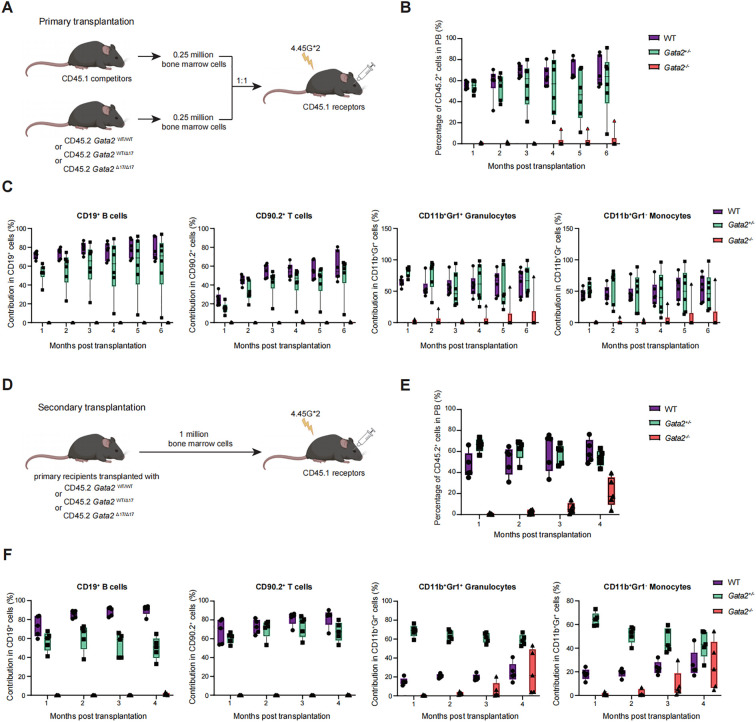
Detection of hematopoietic cells generated in competitive transplantation. **(A)** Scheme of the competitive transplantation experiment. **(B)** Quantification of CD45.2 donor-derived cell proportions in the peripheral blood of primary recipient mice. **(C)** Proportion of CD45.2 donor-derived cells in different hematopoietic lineages including CD19^+^ B cells, CD90.2^+^ T cells, CD11b^+^Gr1^+^ granulocytes, and CD11b^+^Gr1^+^ monocytes in the peripheral blood of primary transplanted mice. n = 5 independent individual mice of one representative experiment. **(D)** Schematic representation of secondary transplantation for the indicated groups. **(E)** Proportion of CD45.2 donor-derived cells in the peripheral blood of secondary transplanted mice. **(F)** Proportion of CD45.2 donor-derived cells in different hematopoietic lineages, including CD19^+^ B cells, CD90.2^+^ T cells, CD11b^+^Gr1^+^ granulocytes, and CD11b^+^Gr1^+^ monocytes in the peripheral blood of secondary transplanted mice. n = 5 independent individual mice of one representative experiment.

Given the reduced repopulation capacity in *Gata2*-deficient BM transplants, which precluded an assessment of the multi-lineage differentiation potential of *Gata2^-/-^* HSCs, we conducted competitive transplantation assays. The WT, *Gata2^+/−^*, and *Gata2^−/−^* BM cells (CD45.2^+^) were mixed with competitor cells (CD45.1^+^) at a 1:1 ratio and then transplanted into lethally irradiated recipients (CD45.1^+^) (**Figure 5A**). Consistent with prior observations, minimal donor-derived chimerism (CD45.2^+^) was detected in recipients transplanted with *Gata2*-deficient BM cells. By the fourth month, only one recipient exhibited low-level chimerism (**Figure 5B**), with donor-derived cells exclusively restricted to granulocyte and monocyte populations, lacking lymphoid lineages (**Figure 5C**). Intriguingly, a progressive increase in CD45.2^+^ chimerism was seen in this recipient at the fifth and sixth months and the myeloid differentiation capacity of *Gata2^-/-^* HSCs in this specific recipients was preserved (**Figures 5B, C**). At six months, the recipient was euthanized for secondary transplantation (**Figure 5D**). Mirroring the primary engraftment pattern, secondary recipients exhibited compromised CD45.2^+^ chimerism (**Figure 5E**) dominated by myeloid cells (granulocytes and monocytes) at 3 and 4 months, with no detectable CD45.2^+^ donor-derived B or T cells (**Figure 5F**). These findings collectively suggest that *Gata2^-/-^* HSCs retain attenuated long-term hematopoietic reconstitution capacity, but their differentiation potential is restricted to the myeloid lineage.

### Transcriptomic profile of the *Gata2* mutated HSCs

**Figure 6:**
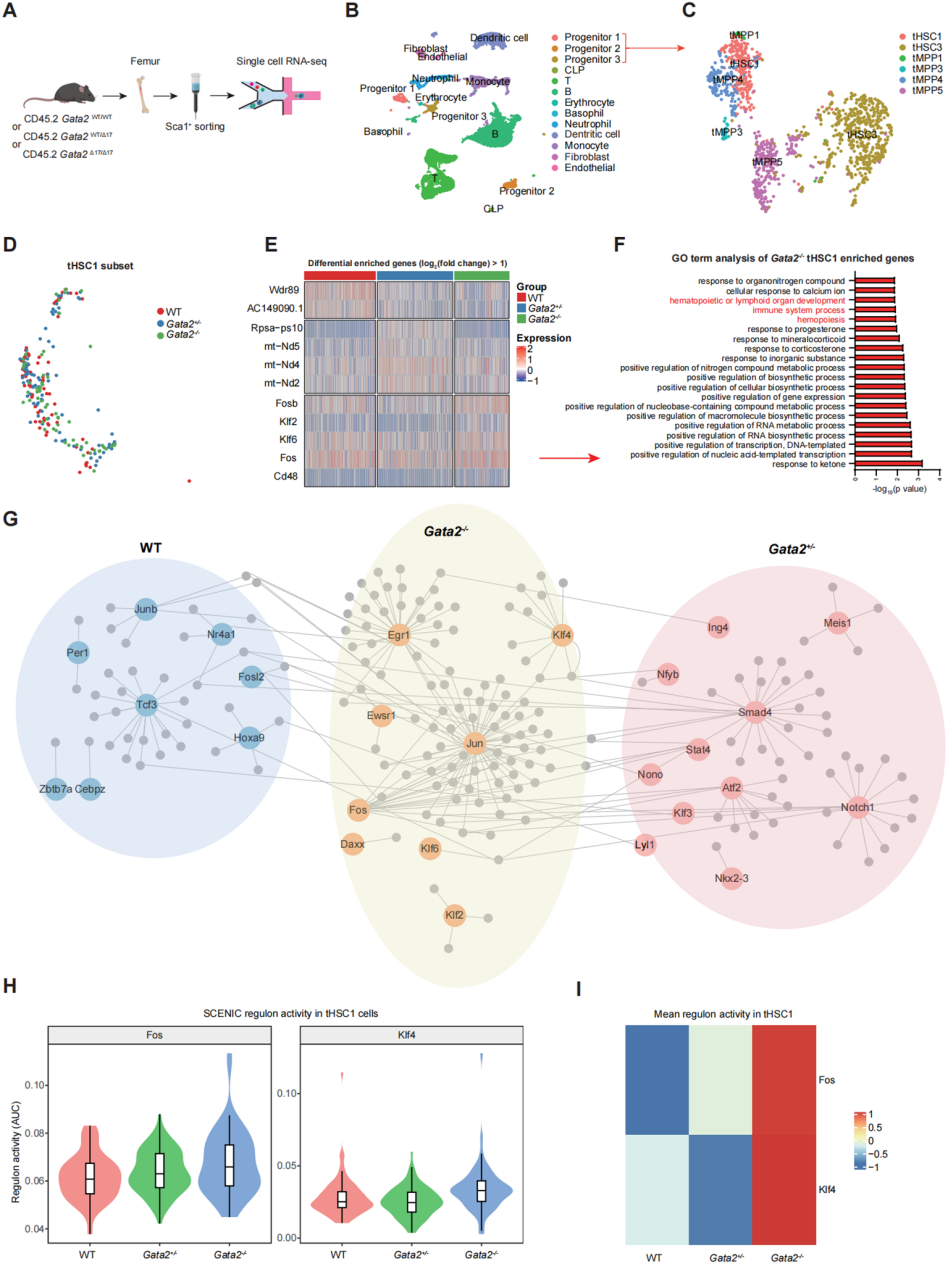
Transcriptome profile of the *Gata2* deficiency HSCs. **(A)** Scheme of scRNA-seq analysis of Sca1^+^ cells from the bone marrow of WT, *Gata2^+/−^*, and *Gata2^−/−^* mice. 1×10^5^ Sca1^+^ cells from each mice were collected were used for single cell capture to prepare scRNA-seq library. N = 3 mice for each group. 21859 single cells were obtained after data infiltration and used for subsequently analysis. n= 10307 for WT group. n= 7759 for *Gata2^+/−^* group. n= 3793 for *Gata2^−/−^* group. **(B)** Unsupervised clustering of single cells. Each cell population is shown in the figure. **(C)** Sub-clustering of hematopoietic progenitor populations from the three progenitor groups. **(D)** tHSC1 cell population detected in the indicated groups. **(E)** Analysis of differentially expressed transcription factors (log_2_(fold change) > 1, p < 0.05) in tHSC1 cells across the of the indicated genotypes. **(F)** Gene ontology (GO) enrichment analysis of the differentially expressed transcription factors. **(G)** Cytoscape display of a regulatory network composed of transcription factors (log_2_(fold change) > 0.25, p < 0.05) and their target genes. The edges connect transcription factor-target gene pairs, while the nodes represent genes. Transcription factors are displayed in a larger font, and the three different groups (WT, *Gata2^+/−^*, and *Gata2^-/-^*) are distinguished using different background colors. **(H)** Violin plots showing the distribution of SCENIC regulon activity scores (AUC) for Fos and Klf4 in tHSC1 cells from WT, *Gata2^+/−^*, and *Gata2^−/−^* samples. Boxplots indicate the median and interquartile range. **(I)** Heatmap showing the mean regulon activity of Fos and Klf4 across WT, *Gata2^+/−^*, and *Gata2^−/−^* tHSC1 cells. Values were row-scaled for visualization to highlight relative differences among groups.

To investigate the properties of *Gata2*-deficient HSCs and the mechanisms underlying HSC emergence, we conducted single-cell RNA sequencing (scRNA-seq) on Sca1^+^ cells enriched from the femoral BM of WT, *Gata2^+/−^*, and *Gata2^−/−^* mice using magnetic bead sorting (**Figure 6A**). Unsupervised clustering identified major hematopoietic lineages, including T cells, B cells, granulocytes, and monocytes, as well as endothelial cells and three distinct hematopoietic progenitor clusters (**Figure 6B**; **Supplementary Figure S4A**). Sub-clustering of progenitors revealed subgroups annotated as tHSC1/3 and tMPP1/3/4/5 (**Figure 6C**; **Supplementary Figure S4B**), with tHSC1 representing the majority of the long-term HSC (LT-HSC) population (25). Notably, tHSC1 populations were detected across all genotypes (**Figure 6D**; **Supplementary Figure S4C**). Consistent with previous findings, transcriptomic analysis of cell cycle states revealed that the three distinct tHSC1 populations exhibited similar cell cycle status at the transcriptional level (**Supplementary Figure 4D**). We next compared tHSC1 transcriptomes across genotypes, focusing on differentially expressed transcription factors (TFs) (**Figure 6E**). Gene ontology (GO) enrichment analysis of these TFs revealed their significant association with hematopoiesis (**Figure 6F**). Strikingly, *Fos—*a TF previously implicated in fibroblast-to-HPC trans-differentiation *in vitro (*26) and HSC generation *in vivo (*27)—was markedly upregulated in *Gata2^-/-^* tHSC1 cells. Network analysis of highly expressed TFs and their target genes in each genotype using Cytoscape (**Figure 6G**) revealed the enrichment of Hoxa9 in WT tHSC1 cells, a key hematopoietic regulator (28, 29), and the enrichment of *Fos*, *Klf4*, and *Klf6* in Gata2^-/-^ tHSC1 cells. Particularly, Klf4, a “Yamanaka factor”, is critical for reprogramming and cell fate determination (30), and Klf6 loss is associated with HSC aging (31). Interestingly, SCENIC analysis identifies *Fos* and *Klf4* as the transcription factor important for tHSC1 (**Figures 6H, I**). This finding suggests that upregulation of *Fos* and other TFs might partially compensate for *Gata2* loss, thereby maintaining the impaired self-renewal and the multi-lineage potential of *Gata2^-/-^* HSCs.

### Transcriptome profile of the *Gata2* mutated T cells

**Figure 7:**
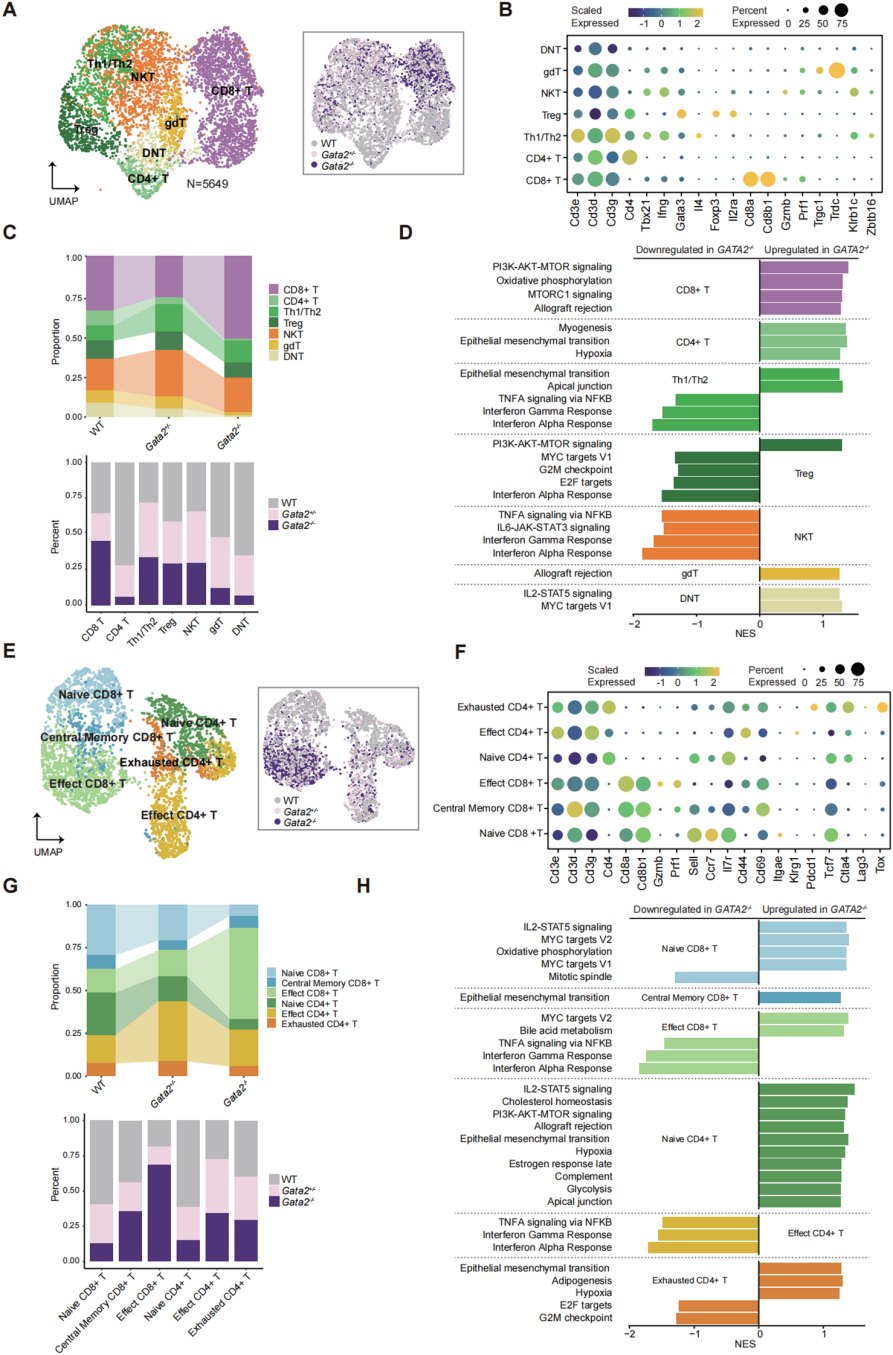
Transcriptome profile of the *Gata2* deficiency T cells. **(A)** UMAP plot of T cell clustering, with clusters colored according to inferred cell types, including CD8^+^ T, CD4^+^ T, Th1/Th2, Treg, NKT, and gdT cell populations. UMAP plots of cells colored by different groups (WT, *Gata2^+/−^*, and *Gata2^-/-^*). **(B)** Dot plot showing cell clusters defined by gene expression of known markers. **(C)** The proportion of different cell populations within the indicated groups, as well as the proportion of each cell population across different groups. **(D)** GSEA revealing significantly altered pathways in *Gata2^-/-^* compared to WT mice. **(E)** T cell populations within CD8^+^ and CD4^+^ T cells are categorized by functional state, including naive CD8^+^ T, central memory CD8^+^ T, effector CD8^+^ T, naive CD4^+^ T, effector CD4^+^ T, and exhausted CD4^+^ T cell groups. UMAP plots of cells colored by different groups (WT, *Gata2^+/−^*, and *Gata2^-/-^*). **(F)** Dot plot displaying cell clusters defined by gene expression of known markers. **(G)** Proportions of various cell populations within different groups, as well as the proportions of each cell population across indicated groups. **(H)** GSEA revealing significantly altered pathways in *Gata2^-/-^* compared to WT.

Building on the observation that *Gata2* deficiency preserves T cell homeostasis in primary lymphoid organs (peripheral blood and thymus), including CD4+/CD8+ single-positive populations, we employed scRNA-seq to map the transcriptional profiles of discrete T cell subsets. Sub-cluster analysis identified functionally distinct T cell subsets, including CD8, CD4, Th1/Th2, regulatory T (Treg), natural killer T (NKT), and γδT (gdT) cells (**Figures 7A, B**). While single-cell analysis confirmed all major T cell subsets in *Gata2*-deficient mice, proportional differences were observed (**Figure 7C**). These were consistent with the inter-individual variation observed in the peripheral blood and thymus T cell cohorts (**Figure 1F**, **3E**), suggesting that *Gata2* deficiency does not induce systematic T cell subset imbalances. GSEA revealed no overt transcriptional remodeling in *Gata2-*deficient T cell subsets (**Figure 7D**).

We further characterized T cell functional heterogeneity by isolating CD4+ and CD8+ T cells and classifying them by canonical functional markers. This revealed distinct subsets including naive, central memory, and effector CD8+ T cells, alongside naive, effector, and exhausted CD4+ T cells (**Figures 7E, F**). All subsets were preserved across all genotypes, albeit with minor proportional variations observed (**Figure 7G**), suggesting that *Gata2* deficiency does not globally disrupt T cell homeostasis. At the transcriptional level, GSEA between *Gata2^−/−^* and WT T cells identified limited pathway enrichments. Specifically, *Gata2^−/−^* downregulation of IFNG/A and TNFA signaling pathways was observed in Th1/Th2 and NKT cells (**Figure 7D**) as well as effector CD4^+^/CD8^+^ T cells (**Figure 7H**), implying impaired antigen-stimulated responses of *Gata2*-deficient T cells. These findings indicate that while *Gata2* loss preserves fundamental T cell subset composition, it may selectively compromise function through dysregulating key immune pathways. Importantly, our data collectively demonstrate that *Gata2* deficiency does not induce global transcriptional remodeling of the T cell landscape.

## Discussion

This study challenges the conventional hematopoietic hierarchy by demonstrating that T-cell development independent of definitive hematopoiesis in adult *Gata2*-deficient mice. While prior studies established *Gata2* as indispensable for HSC generation and maintenance, our identification of viable *Gata2^-/-^* mice with preserved T-cell development, despite profound HSC depletion and defective lymphoid progenitor generation, reveals unexpected T lymphopoiesis independent of canonical definitive hematopoiesis. This finding raises critical questions about the ontogenetic origins and regulatory mechanisms sustaining T-cell development under severe hematopoietic compromise.

The survival of *Gata2^-/-^* HSCs in mice, which exist in survived pups, albeit at extremely low frequencies, demonstrates attenuated hematopoietic repopulation capacity and biased multi-lineage differentiation potential. This suggests the existence of compensatory mechanisms supporting these functions. Single-cell transcriptomic profiling of mutant HSCs revealed the upregulation of key regulators such as *Fos*, *Klf4*, and *Klf6*, which may mitigate the loss of *Gata2*. *Fos*, in particular, has been implicated in HSC generation and the trans-differentiation of somatic cells into hematopoietic progenitors, potentially explaining the residual HSC population generated in viable *Gata2^-/-^* mice and the myeloid reconstitution capacity observed in transplantation assays. These compensatory networks may sustain a minimal HSC pool capable of myeloid output while failing to support lymphopoiesis from these transplanted HSCs, highlighting lineage-specific dependencies on *Gata2*.

The preserved T-cell compartment in *Gata2^-/-^* mice contrasts sharply with the profound B-cell deficiencies observed. In contrast, transplanted *Gata2^-/-^ HSCs* rarely differentiate into T and B cells in recipient mice. This dichotomy aligns with emerging evidence suggesting early embryonic origins of lymphoid potential that precede definitive HSC emergence. While B-cell development strictly requires HSC-derived CLPs, our data imply that T-cell progenitors may arise through alternative pathways. The intact thymic DN-to-SP progression in mutants suggests either (1) residual HSC activity sufficient to seed thymic progenitors, or (2) the existence of Gata2-independent progenitors specifically supporting T-lineage commitment, which originate from embryonic stage and persist to adult life. The latter possibility gains credence from a recent report of HSC-independent lymphoid progenitors persisting from the postnatally stage to adult (32). Further analysis of ligand-receptor interaction between the HSCs, niche cells and T-cell progenitor cells at the embryonic stage may provide clues for whether alternative microenvironmental signals support the T-cell compartment development of HSC-independent lymphoid progenitors. However, this might be challenging due to the extreme low frequency of Gata2^-/-^ pups which can survive embryonic lethality due to Gata2 deficiency. Notably, the splenic expansion of B progenitors in mutants, despite the extremely low frequency of these progenitors in the bone marrow, further underscores the plasticity of hematopoietic niches in compensating for lineage-specific defects. Fate-mapping experiments are needed to determine the origin of the T cells in *Gata2^-/-^* mice.

The skewed myeloid reconstitution capacity of *Gata2^-/-^* HSCs across serial transplants, reinforces the concept of lineage-primed HSC subsets with varying *Gata2* dependencies. The complete absence of donor-derived lymphoid cells in competitive assays suggests that *Gata2* is critical for lymphoid fate decision from HSCs. However, *Gata2^-/-^* HSCs, although with attenuated repopulating capacity, support the generation of myeloid lineages for up to 4 months without competitors, especially the granulocyte lineage. This myeloid features partially explain the clinical manifestations of *GATA2* deficiency syndromes, where thrombocytopenia progresses to myeloid malignancies (33).

Several unanswered questions emerge from these findings. First, the developmental origin of T-cell progenitors in *Gata2^-/-^* mice remains unclear—whether they derive from residual definitive HSCs, embryonic-derived lymphoid precursors, or transdifferentiated cells. Second, whether the phenotypic T cells in the primary *Gata2^-/-^* mice functions normally was not tested. Third, the role of extrathymic microenvironments in sustaining T-cell development warrants investigation, particularly given the splenic compensation observed in B-lineage cells. Fourth, the temporal regulation of compensatory factors like Fos requires elucidation, as their transient versus sustained expression may determine the long-term viability of mutant HSCs. Finally, given that GATA2 mutations could predispose to chromosomal abnormalities (34), it is important to determine whether such abnormalities are involved in these observations made in *Gata2^-/-^* mice.

Also, this study has several technical and biological limitations. First, the extremely low survival rate of *Gata2^-/-^* mice (1.2%) resulted in a small cohort (n = 5), limiting the statistical power to assess phenotypic variability across individuals. A larger cohort of *Gata2^-/-^* mice is needed to further validate the findings in this study. Second, the limited number of mice assayed in the transplantation and single cell analysis raises concerns about sampling bias. Third, the absence of longitudinal data prevents definitive conclusions about whether T-cell preservation reflects transient compensation or sustained adaptation. Fourth, the T cell function in *Gata2^-/-^* mice has not been comprehensively analyzed, such as cytokine production or pathogen clearance. Fifth, while the myeloid bias in mutants parallels human *GATA2* deficiency syndromes, the model fails to recapitulate clinical features such as MDS or AML, highlighting species-specific differences in immune regulation. Finally, both male and female *Gata2^-/-^* mice failed to produce offspring, strongly suggesting an association with infertility. While this phenotype aligns with clinical reports linking *GATA2* deficiency to reproductive dysfunction, we did not uncover the underlying mechanisms (35–37).

In conclusion, this study redefines our understanding of the hematopoietic hierarchy by demonstrating that T-cell development can bypass classical definitive hematopoiesis and persist into adult life. The findings have significant implications for interpreting hematopoietic failure states and designing therapies for *GATA2*-related disorders. By identifying compensatory networks that sustain limited hematopoiesis without Gata2, this work opens new avenues for exploring regenerative strategies targeting alternative regulatory pathways in stem cell biology.

## Data Availability

The datasets presented in this study can be found in online repositories. The names of the repository/repositories and accession number(s) can be found in the article/**Supplementary Material**.
