# Supplementary material for: Viable Gata2 deficient mice exhibit T cell development independent of adult definitive hematopoiesis
Source: Front Immunol. 2026 May 22;17:1832484. doi: 10.3389/fimmu.2026.1832484 (PMC13236849; doi:10.3389/fimmu.2026.1832484)

Supplementary Files

for "**Viable Gata2 deficient mice exhibit T cell development independent of adult definitive hematopoiesis**"

Supplementary Figures

Supplemental Figure 1 Gata2 gene deletion and peripheral blood cell count results in Gata2-deficient mice.
Supplemental Figure 2 Analysis of hematopoietic stem cells in bone marrow of the indicated groups.
Supplemental Figure 3 Comparison of spleen and its cells of the indicated groups.
Supplemental Figure 4 Single-cell transcriptomic gene dot plot analysis, cell cluster analysis and cell cycle analysis.

Supplemental Figure Legend

Supplemental Figure 1 *Gata2* gene deletion and peripheral blood cell count results in Gata2-deficient mice. (**A**) Schematic representation of 17 nucleotide deletion in Gata2 gene and resultant truncated protein in mice. (**B**) Sequencing validation of Gata2 gene in mouse peripheral blood and organs. **(C)** Cell count of whole blood cell (WBC), red blood cell (RBC) and platelet (PLT) of the indicated mice.

Supplemental Figure 2 Analysis of hematopoietic stem cells in bone marrow of the indicated groups. (**A**) Bone marrow mononuclear cell (BMNC) counts of the indicated groups. Flow cytometry analysis (**B**) and statistics (**C**) of HSPC subset classification, including HSC^{LT}, HSC^{ST} and MPP based on CD135 and CD34 expression. *, $p<0.05$; **, $p<0.01$; ***, $p<0.001$; ns, no significance. (**D**) Flow cytometry analysis of HSC proportions, cell cycle, and apoptosis in the bone marrow of the indicated groups. (**E**) Statistics of HSC cell cycle distribution of the indicated groups. ns, no significance. (**F**) Statistics of apoptosis levels in HSCs of the indicated groups. ns, no significance. (**G**) Colony-forming unit (CFU) potential of BMNCS the indicated groups.

Supplemental Figure 3 Comparison of spleen and its cells of the indicated groups. (**A**) Schematic diagram of common lymphoid progenitor (CLP) development. (**B**) Comparison of spleen size between WT, *Gata2*$^{+/-}$ and *Gata2*$^{-/-}$ mice. (**C**) Statistical analysis of spleen weight in the indicated mice. *, $p<0.05$; **, $p<0.01$; ns, no significance. (**D**) Flow cytometry analysis of the B cell progenitors in the spleen (CD45.2^{+}Lin- gated), including B220^{+}CD43^{-} pre-B cells and B220^{+}CD43^{+} pro-B cells. The pro-B cells were further divided into BP-1^{-}CD24^{-} pre-pro-B cells, BP-1^{-}CD24^{+} early pro-B cells and BP-1^{+}CD24^{+} late-pro-B cells.

Supplemental Figure 4 Single-cell transcriptomic gene dot plot analysis, cell cluster analysis and cell cycle analysis. (**A**) Dot plot showing cell clusters denoted by gene expression of known markers. (**B**) Dot plot depicting three progenitor groups in cell clusters identified by expression of known marker genes. (**C**) Proportion of each cell type across the indicated groups. (**D**) Cell cycle profiles of tHSC1 populations across genotypes.

Supplemental Figure 1

A

Gata2-WT (480aa in length)
aa sequence:
MEVAPEQPRWMAHPAVLNAQHPDSHHPGLAHNYMEPAQLL......

Gata2$^{-/-}$ (Δ17/Δ17) (25aa in length)
aa sequence:
MEVAPEQPRWIECAAPRLAPSGPGA*

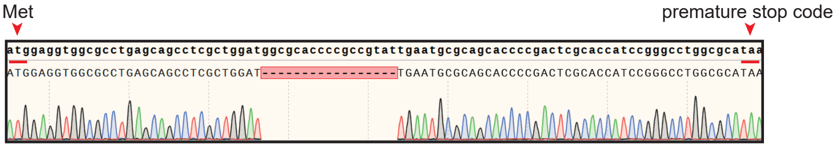

B

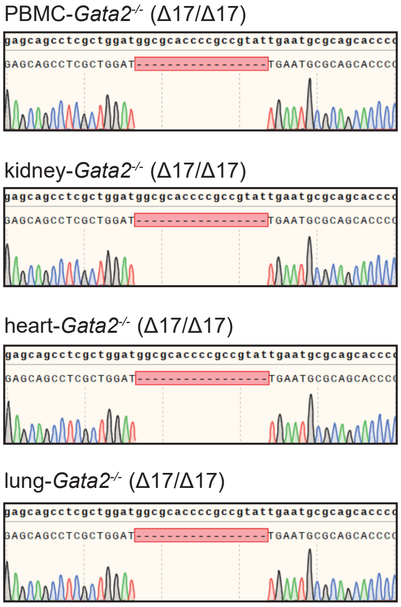

C

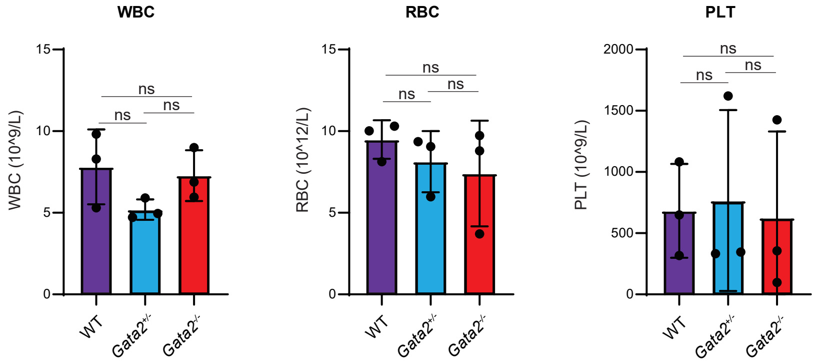

Supplemental Figure 2

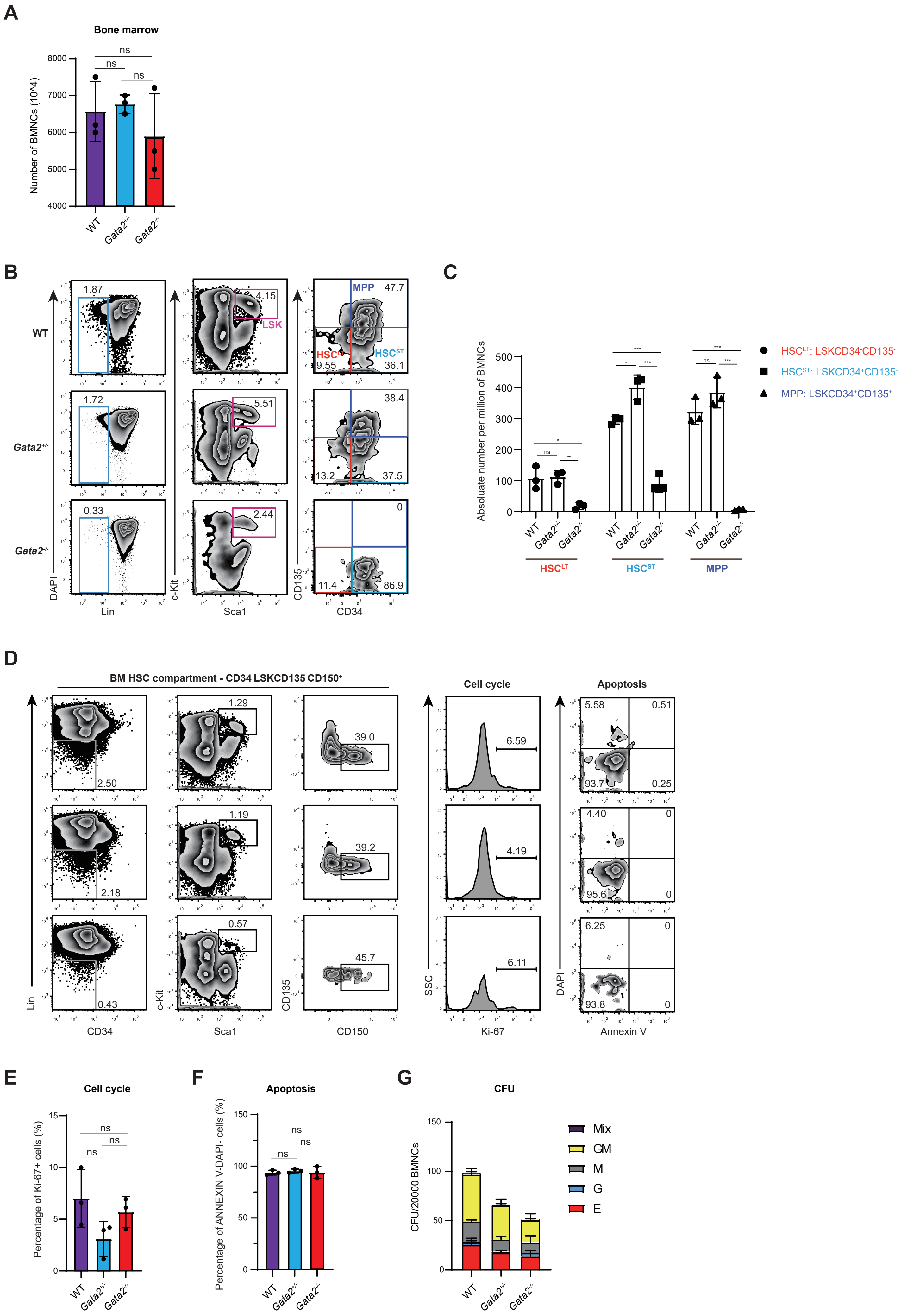

Supplemental Figure 3

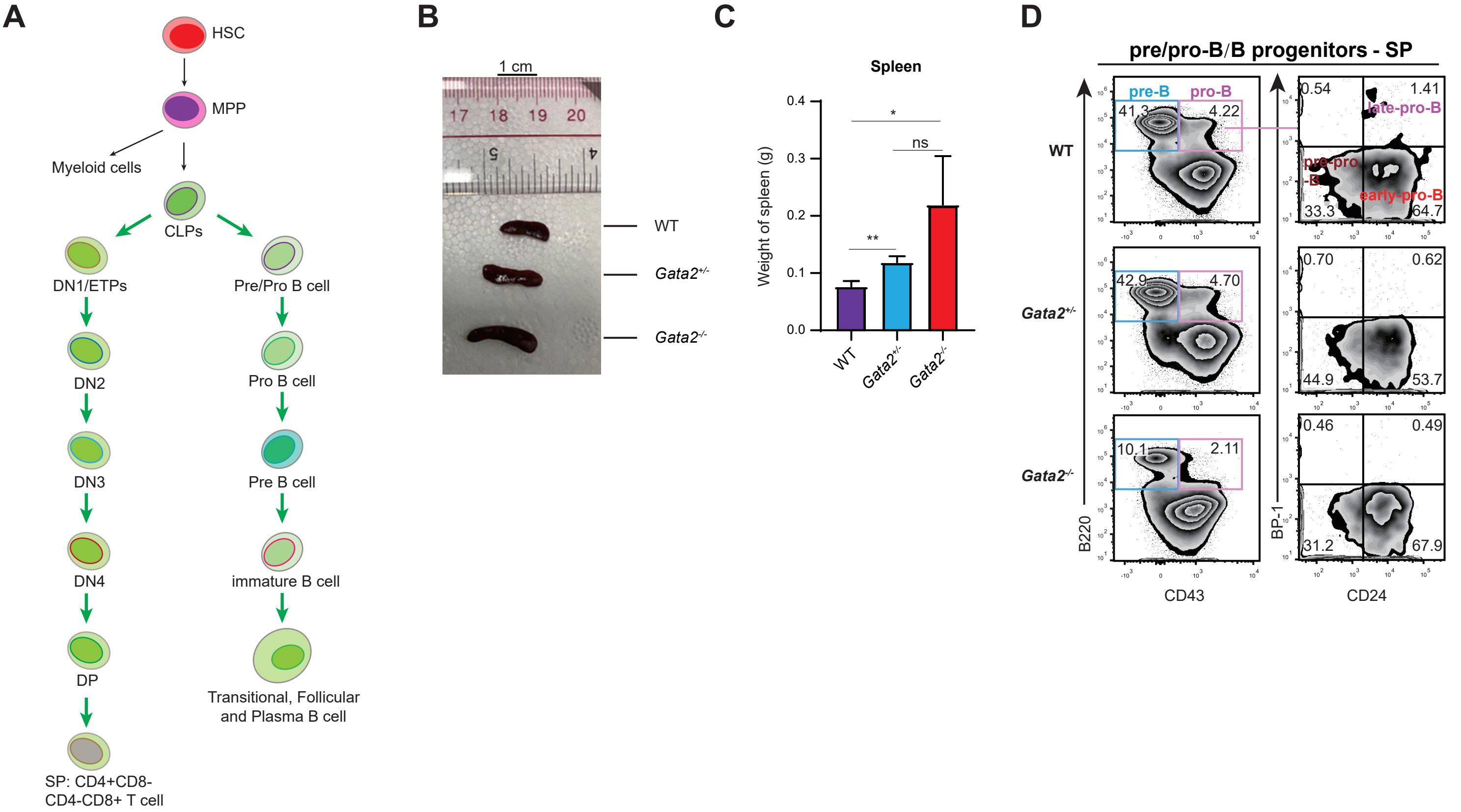

Supplemental Figure 4

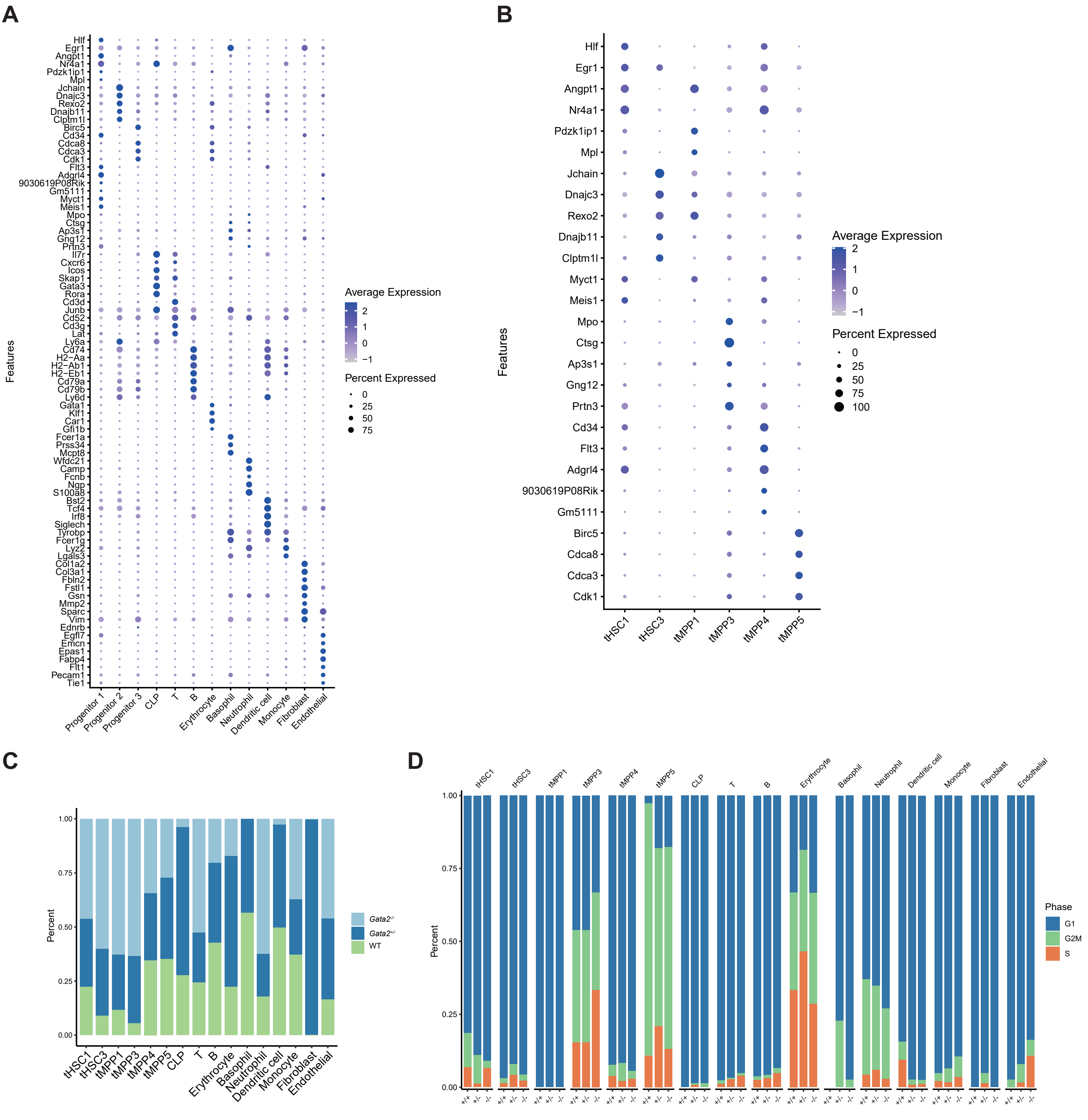

Supplement: Supplementary file 1 [file Image1.pdf]
